# Supplementary material for: Overexpression of BDNF and uPA Combined with the Suppression of Von Hippel–Lindau Tumor Suppressor Enhances the Neuroprotective Activity of the Secretome of Human Mesenchymal Stromal Cells in the Model of Intracerebral Hemorrhage
Source: Int J Mol Sci. 2025 Jul 12;26(14):6697. doi: 10.3390/ijms26146697 (PMC12294669; doi:10.3390/ijms26146697)
Supplement: Supplementary file 1 [file ijms-26-06697-s001.zip › ijms-3705892-supplementary.pdf]

Table S1. Primer sequences and amplification parameters used in the study.

Gene	Nucleotide sequence (5′ -> 3′)	Annealing T, ⁰C	Amplicon length, bp
*BDNF (cDNA)**	TTTTTGCTAGCTAGCCACCACCATGGCGAC<br>CATCCTTTTCCTTCTTACTATGGTTATTATTTCATACTTTTTGG<br>TATTAAGCTTTTTTCATCTTCCCCTTTTAATGGTCAATGTACATA<br>CACACAAGAAGTGTGTCTATC	63	771
*uPA (cDNA)**	TTTAGGTAGGTACCAGAGA<br>GAGCTGCTGCTGGCGCGCGCCTGCTGCTTCTTCTCTCTGC<br>TATCGCGCGGGGGGGGGGGGGGGGGGGGGGGGGGGGGGGG<br>GGGGGGGGCCATTATTCTCTTCTTCCTTCTTGGTGTGAC	68	1315
BDNF (qPCR)	TTAGGGGGGTCAAGGTGGGTGGGCTA<br>AGGCTCCAAGGGAAGGGAACTTGTGTG	57	236
uPA (qPCR)	ATTGCGCGACTGCCTGAATGGA<br>TCCGACACCTGCACATAACACC	58	326
VEGFA (qPCR)	ATCAAACCTCACCAAGGCCAG<br>AGGCCCACACAGGGAACGC	58.5	183
HGF (qPCR)	ACCACACCACCGGCACAAATTCT<br>ATCCCAACGCTGACATGGAAT	58	274
36B4 (qPCR)	CGACCTGGAAGTCCAACTAC<br>ATCTGCTGCTGCATCTGCTGCTTG	53	109
VHL (qPCR)	ACACACACGATGGATGGGCTTCTGG<br>GATGTGTGCAATGCGCTCCTG	58	256
shRNA (synthesis)	CACCGAAACACACAGTCCAGGCTACTCCATCACACAGCAT<br>GGAGTAGTAGCCTGGCTGGACTGTTTCCAAAAAAAAAAAAAA<br>AAAAAAAAAAAAAAAAAAAAAAAAAAAAAAAAAAAA<br>AAAAAAAAAAAAAAAAAAAAAAAAAAAAATCCATTCCATTGG<br>TGGTGGTAGGACCAGAGAGCACAGCACAGCCTCTCTCTTG<br>GTCCTTCCTACCAATGGATTCCTTTTTTTTTTTTTTGTTTTTTT	-	-
shRNA (qPCR)	TGGTCCTACCTACCAATGGATTTTTTTTTTTTTTTTTTT<br>TTTTTTTTTTTTTTTTTTTTTTTTTTTTTTTTTTTTTTTTTTTTTTT<br>TTTTTTTTTTTTTTTT<br>AAACAAAAAAAAAAAGGAAAGGAAATCCCCATTGGATTGG-<br>TAGGACCA	61	156

* - estimated annealing temperature for the use of PCR mixtures containing Taq, Pfu polymerases, taking into account the degree of matrix complementarity
